# Teeth in Inherited Syphilis

**Published:** 1888-03

**Authors:** 


### Teeth in Inherited Syphilis.

Mr. J. Hutchinson, Jr., at a recent meeting of the Harveian Society, showed patients and drawings illustrating the malformations of the teeth in inherited syphilis. The chief points discovered by Mr. Hutchinson, Sr., were recapitulated, and the fallacy of looking for characteristic signs in the temporary teeth pointed out. Those teeth which in after life showed syphilitic deformity in the greatest degree were those which calcified first, and which during the year lay the nearest to the gum surface—namely, the permanent incisors and first molars. The three small projections seen in normal teeth at the cutting edge of the central incisors were noticed, and the wearing away or non-development of the median one was described in connection with the formation of the semi-lunar notch and the narrow cutting edge. The horizon erosion of enamel from mercurial stomatitis in infancy and its occasional coincidence with true syphilitic malformation were illustrated, and stress laid on the fact that the upper central incisors were the real test teeth of inherited syphilis, though in many cases all the teeth were normal.

